# Ranakinestatin-PPF from the Skin Secretion of the Fukien Gold-Striped Pond Frog, *Pelophylax plancyi fukienensis*: A Prototype of a Novel Class of Bradykinin B_2_ Receptor Antagonist Peptide from Ranid Frogs

**DOI:** 10.1155/2014/564839

**Published:** 2014-04-09

**Authors:** Jie Ma, Yu Luo, Lilin Ge, Lei Wang, Mei Zhou, Yingqi Zhang, Jinao Duan, Tianbao Chen, Chris Shaw

**Affiliations:** ^1^Natural Drug Discovery Group, School of Pharmacy, Queen's University, Belfast BT9 7BL, UK; ^2^The First Hospital of Hebei Medical University, Shijiazhuang 050031, China; ^3^Jiangsu Key Laboratory for Traditional Chinese Medicine (TCM) Formulae Research, Nanjing University of Chinese Medicine, Nanjing, China

## Abstract

The defensive skin secretions of many amphibians are a rich source of bradykinins and bradykinin-related peptides (BRPs). Members of this peptide group are also common components of reptile and arthropod venoms due to their multiple biological functions that include induction of pain, effects on many smooth muscle types, and lowering systemic blood pressure. While most BRPs are bradykinin receptor agonists, some have curiously been found to be exquisite antagonists, such as the maximakinin gene-related peptide, kinestatin—a specific bradykinin B_2_-receptor antagonist from the skin of the giant fire-bellied toad, *Bombina maxima*. Here, we describe the identification, structural and functional characterization of a heptadecapeptide (DYTIRTRLHQGLSRKIV), named ranakinestatin-PPF, from the skin of the Chinese ranid frog, *Pelophylax plancyi fukienensis*, representing a prototype of a novel class of bradykinin B_2_-receptor specific antagonist. Using a preconstricted preparation of rat tail arterial smooth muscle, a single dose of 10^−6^ M of the peptide effectively inhibited the dose-dependent relaxation effect of bradykinin between 10^−11^ M and 10^−5^ M and subsequently, this effect was pharmacologically-characterized using specific bradykinin B_1_- (desArg-HOE140) and B_2_-receptor (HOE140) antagonists; the data from which demonstrated that the antagonism of the novel peptide was mediated through B_2_-receptors. Ranakinestatin—PPF—thus represents a prototype of an amphibian skin peptide family that functions as a bradykinin B_2_-receptor antagonist herein demonstrated using mammalian vascular smooth muscle.

## 1. Introduction

Bradykinin (BK) is a nonapeptide with the primary structure, RPPGFSPFR. It is a peptide with a wide spectrum of biological effects that have been most extensively studied in mammals [1, 2]. A major physiological role for the peptide in mammals, including humans, is in the maintenance of systemic blood pressure through its role in dilation of arterial smooth muscle that effectively counteracts the constrictor effects of angiotensin-II [3–5]. This vasoregulatory role has been exploited clinically through the introduction of inhibitors to the angiotensin-II generating/bradykinin-degrading protease, angiotensin-converting enzyme (ACE), that still represent the front-line therapeutics for the treatment of hypertension [6, 7]. Of major interest to the peptide chemist/biologist is that the lead compounds for the design of ACE inhibitors—a milestone in twentieth century drug discovery—was a family of so-called bradykinin-potentiating peptides (BPPs) from the venom of the South American pit viper,* Bothrops jararaca* [8]. Such BPPs are now known to be of widespread occurrence in snake venoms and analogs have recently been reported from the defensive skin secretions of phyllomedusine frogs [9].

Bradykinins and related peptides (BRPs) are known to be widespread and abundant components of many venoms and defensive secretions and are particularly diverse in structure within the skin secretions of amphibians [10, 11]. This widespread occurrence across taxa is not surprising in view of their wide spectrum of biological effects, not the least of which are their potent algesic effects [10, 11].

Amphibian skin BRPs appear to reflect the primary structures of their endogenous counterparts in vertebrate predator taxa as analogs to most of these amphibian BRPs have been found to be generated from respective plasma proteins by appropriate chemical or physical treatments [10, 11].

While most amphibian BRPs have agonist properties following interaction with appropriate taxon-specific receptors in sourced tissues, some of these, and indeed other structurally unrelated peptides, have been found to have mammalian BK receptor antagonist properties [10, 11]. The skin secretions from bombinid toads appear to be a rich source of receptor agonist BRPs but also of these structurally diverse and structurally unrelated BK receptor antagonist peptides [12–17]. The skin secretions of ranid frogs, however, have proven to be the richest amphibian source of BRPs [10, 18] yet, to date, they remain poorly evaluated for the presence of BK receptor antagonists.

Here, we describe the identification of a bradykinin inhibitory heptadecapeptide, named ranakinestatin-PPF, in the skin secretion of a ranid frog, the Fukien gold-striped pond frog,* Pelophylax plancyi fukienensis*. The peptide was first isolated through its inhibitory effects on bradykinin-induced dilation of a rat tail artery smooth muscle preparation and was subsequently subjected to preliminary structural characterization using MS/MS fragmentation sequencing. Using this sequence data to design a degenerate PCR primer, the cDNA encoding its biosynthetic precursor was successfully cloned from a skin secretion-derived cDNA library, translation of which established the peptides unequivocal primary structure. Pharmacological characterization of a synthetic replicate of the peptide, using the rat tail artery smooth muscle preparation, confirmed its functional bradykinin inhibitory effects and established its probable molecular target as the bradykinin B_2_-receptor. Ranakinestatin-PPF thus represents the prototype of a novel family of bradykinin B_2_-receptor antagonists from the skin secretions of ranid frogs.

## 2. Materials and Methods

### 2.1. Specimen Biodata and Secretion Harvesting

Adult specimens of the Fukien gold-striped pond frog,* Pelophylax plancyi fukienensis* (*n* = 3, snout-to-vent length 7 cm), were captured in paddy fields around Fuzhou City, Fujian Province, People's Republic of China (Fukien is the ancient Chinese name for Fujian). Secretion harvesting was performed in the field after which the frogs were released unharmed. Skin secretion was obtained from the dorsal skin using gentle transdermal electrical stimulation as previously described [19]. The stimulated secretions were washed from the skin using deionized water and divided into either 0.2% (v/v) aqueous trifluoroacetic acid (for subsequent peptide characterization) or into cell lysis/mRNA stabilization buffer (Dynal) for subsequent cDNA library construction. Sampling of skin secretion was performed by MZ under a UK Animal (Scientific Procedures) Act 1986, project licence issued by the Department of Health, Social Services and Public Safety, Northern Ireland, and subsequent pharmacological experiments using rat tissues were performed by MZ under a similar personal licence issued by the same authority. All procedures on animals had additionally been vetted by the IACUC of Queen's University Belfast and approved.

### 2.2. Reverse Phase-HPLC Fractionation of Skin Secretion

The acidified skin secretion washings were clarified of microparticulates by centrifugation (13,000 ×g for 10 min) and the decanted clear supernatant was subjected to reverse-phase HPLC fractionation using a Thermoquest gradient HPLC system fitted with a Jupiter semipreparative C-18 column (1 × 25 cm). This was eluted with a linear gradient formed from 0.05 : 99.95 (v/v) trifluoroacetic acid (TFA)/water to 0.05 : 19.95 : 80.00 (v/v/v) TFA/water/acetonitrile in 240 min at a flow rate of 1 mL/min. Fractions (1 mL) were collected at minute intervals and the effluent absorbance was continuously monitored at *λ*214 nm. Samples (100 *μ*L) were removed from each fraction in triplicate and lyophilized and stored at −20°C prior to smooth muscle pharmacological analysis.

### 2.3. Bioactivity Screening Using Arterial Smooth Muscle

Female albino Wistar rats (200–250 g) were euthanized by asphyxiation followed by cervical dislocation. The tail skin was removed and the major tail artery was identified and moistened with Krebs' solution (118 mM NaCl, 4.7 mM KCl, 25 mM NaHCO_3_, 1.15 mM NaH_2_PO_4_, 2.5 mM CaCl_2_, 1.1 mM MgCl_2_, and 5.6 mM glucose) prior to dissection of the proximal region that then placed in ice-cold Kreb's solution. Two mm wide rings of this tissue were cut and mounted onto a transducer prior to placing in 2 mL organ baths containing Kreb's solution (37°C) flowing at 2 mL/min with constant gentle bubbling of 95% O_2_/5% CO_2_. These rings of muscle were equilibrated for 1 h before experimental procedures were initiated. Preparations were perfused constantly with 1 × 10^−5^ M phenylephrine in Kreb's solution in order to obtain constriction plateaux and following this, relative relaxation events were recorded following applications of reconstituted 100 *μ*L samples from HPLC fractions (#1–#240) of* Pelophylax plancyi fukienensis* skin secretion. This was carried out to exclude those fractions (25) containing direct arterial smooth muscle relaxant activity. Following this, the remaining fractions (215) were employed in the following procedure for identification of bradykinin inhibitory peptides. The preparations were incubated as before, with phenylephrine (1 × 10^−5^ M) until constriction plateaux were obtained. Following this, combinations of phenylephrine (1 × 10^−5^ M) and reconstituted nonmyoactive fractions were applied to individual preparations and incubated for 20 min. After this period, a single dose of bradykinin (1 × 10^−6^ M) was added to the medium in each organ bath. Changes in arterial smooth muscle tone were measured by a pressure transducer system and the relaxations induced by bradykinin in the presence of reconstituted fractions were calculated using the PowerLab computer package.

### 2.4. Structural Characterization of the Bradykinin Inhibitory Peptide

Reverse phase HPLC fraction #100 was found to possess bradykinin inhibitory activity in the arterial smooth muscle preparation and a sample of this fraction was subjected to tandem MS/MS fragmentation sequencing using an LCQ-Fleet electrospray ion-trap mass spectrometer (Thermo Fisher, San Jose, CA, USA).

### 2.5. Molecular Cloning of the Bradykinin Inhibitory Peptide Biosynthetic Precursor-Encoding cDN

Polyadenylated mRNA was isolated from the stabilization buffer/skin secretion mixture using magnetic oligo-dT beads as described by the manufacturer (Dynal Biotech, UK) and it was reverse transcribed. The isolated mRNA was then subjected to 5′- and 3′-rapid amplification of cDNA ends (RACE) procedures to obtain full-length bradykinin inhibitory peptide precursor nucleic acid sequence data using a SMART-RACE kit (Clontech UK) as per manufacturer's instructions. Briefly, the 3′-RACE reactions employed a nested universal (NUP) primer (supplied with the kit) and a degenerate sense primer (S: 5′-GAYTAYACIHTIMGIACIMGIHT-3′) (Y = C/T, I = deoxyinosine, H = A/T/C, M = A/C) that was complementary to the N-terminal amino acid sequence, D-Y-T-I/L-R-T-R-L/I-, of the bradykinin inhibitory peptide. The 3′-RACE reactions were purified and cloned using a pGEM-T vector system (Promega Corporation) and sequenced using an ABI 3100 automated DNA sequencer. The sequence data obtained from the 3′-RACE products were used to design a specific antisense primer (AS: 5′-CCACATCAGATGACTTCCAAATGAT-3′) to a defined conserved site within the 3′ nontranslated region of the bradykinin inhibitory peptide-encoding transcripts. 5′-RACE was carried out using this primer in conjunction with the NUP primer and resultant products were purified, cloned, and sequenced.

### 2.6. Chemical Synthesis of the Bradykinin Inhibitory Peptide

Once the unequivocal primary structure of the bradykinin inhibitory peptide had been established through a combination of MS/MS fragmentation sequencing and translation from cloned precursor-encoding cDNA, a synthetic replicate was produced using solid-phase Fmoc chemistry on a PS3 automated solid-phase synthesizer (Protein Technologies, Inc., AZ, USA). The authenticity of the peptide structure was confirmed by MS/MS fragmentation sequencing following reverse phase HPLC purification of cleaved and deprotected synthesis mixture.

### 2.7. Pharmacological Characterization of Synthetic Bradykinin Inhibitory Peptide Using Rat Tail Artery Smooth Muscle

Rat tail artery smooth muscle preparations were prepared for experimental procedures as previously described. Two series of experiments were performed to address the quantitative pharmacological characterisation of the novel bradykinin inhibitory peptide.

1. The inhibitory effect of the peptide at a single pretreatment concentration of 10^−6^ M was assessed on the dose-dependent relaxation of the artery smooth muscle preparations induced by bradykinin in the concentration range of 10^−11^–10^−5^ M.
2. The effects of pretreatment of artery smooth muscle preparations with the specific bradykinin B_1_ receptor antagonist (desArg HOE 140) or the bradykinin B_2_ receptor antagonist (HOE 140) (Sigma-Aldrich, UK) were assessed to compare with those obtained with the bradykinin inhibitory peptide.

Firstly, the stabilized preparations were exposed to bradykinin in the concentration range of 10^−11^–10^−5^ M with and without pretreatment with the bradykinin inhibitory peptide at a single dose of 1 × 10^−6^ M. For these experiments, the bradykinin inhibitory peptide was added to the organ bath once a stable plateau of phenylephrine-induced constriction had been obtained and after a period of 10 min, bradykinin dose-response curves (10^−11^–10^−5^ M) were constructed.

In a second series of experiments, the specific bradykinin receptor antagonists were employed likewise at a single dose (3 × 10^−7^ M) prior to the procedure described above.

During all of these experiments, changes in tension of the arterial smooth muscle preparation were detected by pressure transducers connected to a PowerLab System (AD Instruments Pty Ltd.). Data from six consecutive experiments with each treatment were analyzed to obtain the mean and standard error of responses by Student's *t*-test using the Graph Pad Prism program and dose-response curves were constructed using a best-fit algorithm through the data analysis package provided. Responses were plotted as tension changes in grams against final molar concentrations of the bradykinin inhibitory peptide present in the organ baths.

## 3. Results

### 3.1. Reverse Phase HPLC Fractionation of Skin Secretion and Preliminary Bradykinin Inhibitory Activity Screening

**Figure 1:**
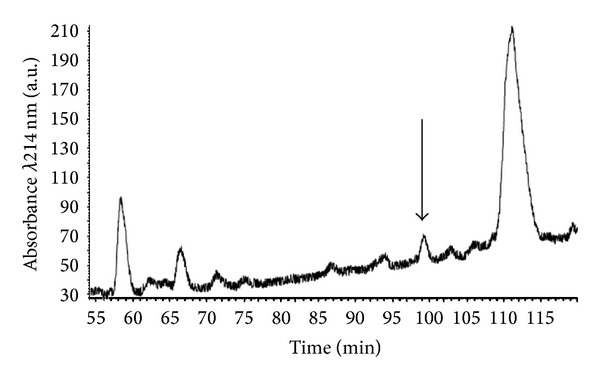
Region of reverse phase HPLC chromatogram of* Pelophylax plancyi fukienensis* skin secretion indicating elution position/retention time of the peak of absorbance corresponding to bradykinin inhibitory activity (arrow).

Reverse phase HPLC fractionation of* P. plancyi fukienensis* skin secretions resulted in the resolution of many components indicating a high degree of molecular complexity. Preliminary screening of samples of each fraction using rat tail artery smooth muscle identified 25 fractions that contained direct vasorelaxant activity and these were excluded from the next series of bioassays that sought fractions with bradykinin inhibitory activity. These fractions were those that could effectively reduce the vasorelaxant action of a single dose (10^−6^ M) of bradykinin. Fraction #100 was found to be effective in this respect and the appropriate region of the reverse phase HPLC chromatogram of skin secretion in which this peptide eluted is shown in Figure 1.

### 3.2. Structural Characterization of the Bradykinin Inhibitory Peptide

**Figure 2:**
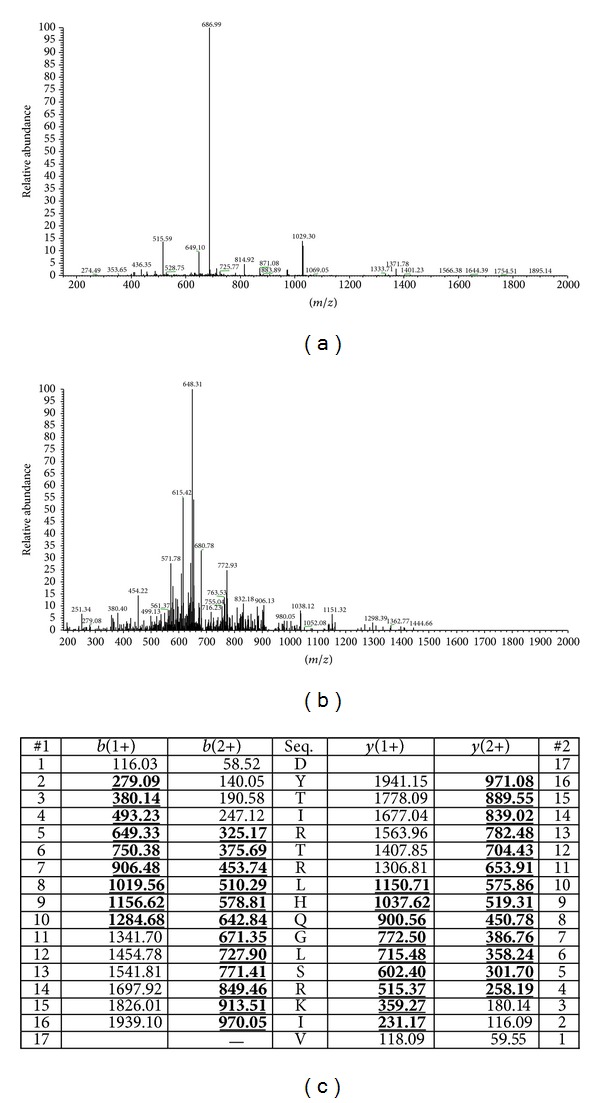
(a) Electrospray MS spectrum of a sample from reverse phase HPLC fraction #100 that contained bradykinin inhibitory activity. The doubly charged ion (*m/z* 1029.30), triply charged ion (*m/z* 686.99), and quadruply charged ion (*m/z* 515.59) of a peptide with a parent mass of 2056.4 Da were detected. (b) MS/MS fragmentation spectrum of the doubly charged ion at* m/z* 1029.30. (c) Theoretical singly and doubly charged* b*- and* y*-ion series arising from MS/MS fragmentation of the bradykinin inhibitory peptide with observed ions indicated in bold typeface and underlined.

A sample of skin secretion reverse phase HPLC fraction #100 was removed and subjected to MS analysis using the electrospray ion-trap mass spectrometer. The sample was found to contain a single major peptide of mass 2056.4 Da, deduced from its doubly charged ion (Figure 2(a)). MS/MS fragmentation sequencing of this doubly charged ion (M + 2H)^2+^,* m/z* 1029.3 (Figure 2(b)), produced the following sequence: DYTL/IRTRL/IHQGL/ISRKL/IV (Figure 2(c)).

### 3.3. Molecular Cloning of the Bradykinin Inhibitory Peptide Biosynthetic Precursor-Encoding cDNA and Bioinformatic Analyses

**Figure 3:**
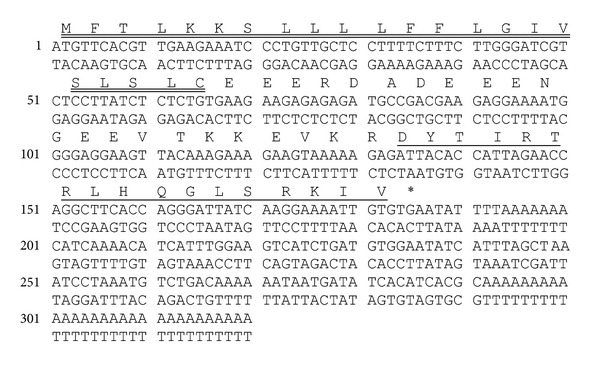
Nucleotide and translated open-reading frame amino acid sequence of cloned* Pelophylax plancyi fukienensis* skin secretion-derived cDNA encoding the biosynthetic precursor of the bradykinin inhibitory peptide, named ranakinestatin-PPF. The putative signal peptide id double-underlined, the mature peptide is single-underlined and the stop codon is indicated with an asterisk.

**Figure 4:**
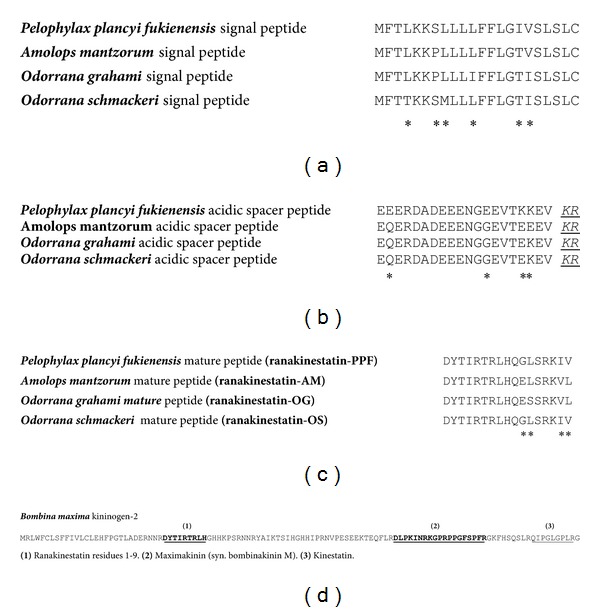
Bioinformatic analysis of the ranakinestatin-PPF biosynthetic precursor with homologs reported from other amphibian sources. Accession numbers for cited sequences are* Pelophylax plancyi fukienensis* ranakinestatin-PPF (HG518554),* Amolops mantzorum* antimicrobial peptide mantzorumin-B_1_ (ADM34242),* Odorrana grahami* odorranaopin (ADP37000),* Odorrana schmackeri* ranakinestatin-OS (HG518555), and* Bombina maxima* skin kininogen-2 (P83055). (a) Comparison of putative N-terminal signal peptide domains of respective biosynthetic precursors. Sites of amino acid differences are indicated with asterisks (6/22). (b) Comparison of acid spacer peptide domains of respective biosynthetic precursors. Sites of amino acid differences are indicated with asterisks (4/20). Note that this domain terminates in the conserved basic amino acid residue doublet (-KR-) (indicated in italics and underlined) that represents the site of propeptide convertase cleavage generating the mature peptide. (c) Comparison of mature (ranakinestatin) peptide domains of respective biosynthetic precursors. Sites of amino acid differences are indicated with asterisks (4/17). (d) The full-length sequence of* Bombina maxima* skin kininogen-2 indicating domains containing (1) the fully conserved ranakinestatin residues 1–9, (2) the bradykinin receptor agonist peptide, maximakinin (syn. bombinakinin M), and (3) the bradykinin B_2_-receptor antagonist peptide, kinestatin.

The molecular cloning strategy adopted for designing a degenerate primer pool to the N-terminal 8 residues of the bradykinin inhibitory peptide for use in 3′-RACE PCR proved to be successful. A single transcript was consistently obtained and was of identical nucleotide sequence in at least 25 different clones. The nucleotide and translated open-reading frame amino acid sequence of this is shown in Figure 3. The open-reading frame encoding the bradykinin inhibitory peptide precursor consisted of 61 amino acid residues with a domain architecture that was consistent with that observed for the majority of amphibian skin peptide precursors. The first domain was constituted by a putative 22-residue signal peptide followed by a 20-residue acidic spacer peptide that immediately preceded a classical-KR-propeptide convertase processing site upstream of a mature peptide encoding domain (Figure 4). From this translated cloned cDNA template, the unequivocal primary structure of the mature bradykinin inhibitory peptide was established as DYTIRTRLHQGLSRKIV (2056.38 Da). The C-terminal residues of the mature peptide domain (and precursor) were IV, indicating that the mature peptide was not C-terminally amidated. The peptide was named ranakinestatin-PPF in accordance with previously established nomenclature. Kinestatin was a prototype bradykinin B_2_-receptor antagonist peptide from amphibian skin, that of the bombinid toad,* Bombina maxima* [15], hence, the adoption of this functional name with the addition of the prefix,* Rana*. The suffix, -PPF, is an abbreviation of the species (and subspecies) of origin,* Pelophylax Plancyi Fukienensis*. This provides the basis for the future systematic classification of additional homologs from other species. Bioinformatic analysis of the ranakinestatin-PPF precursor using the US National Center for Biotechnological Information (NCBI) online database and the BLAST program found two archived homologs. The three domains of these homologs are aligned with those of the ranakinestatin-PPF precursor domains in Figures 4(a)–4(c). These ranakinestatin-PPF homologs are both from ranid frogs, the antimicrobial peptide mantzorumin-B_1_, from the skin of* Amolops mantzorum* (accession no. ADM34242) and the antinociceptive peptide odorranaopin, from the brain of* Odorrana grahami* (ADP37000) [20]. Also included in this comparison are the biosynthetic precursor domain sequences of ranakinestatin-OS (*Odorrana schmackeri*), deduced from cloned skin secretion-derived cDNA (unpublished data). Of particular interest, illustrated in Figure 4(d), is the presence of a sequence identical to the fully conserved N-terminal nonapeptide of the ranakinestatins, within the N-terminal domain of the* Bombina maxima* skin kininogen-2 precursor, that also encodes maximakinin and kinestatin [15].

### 3.4. Pharmacological Characterization of Synthetic Ranakinestatin-PPF Peptide Using Rat Tail Artery Smooth Muscle

**Figure 5:**
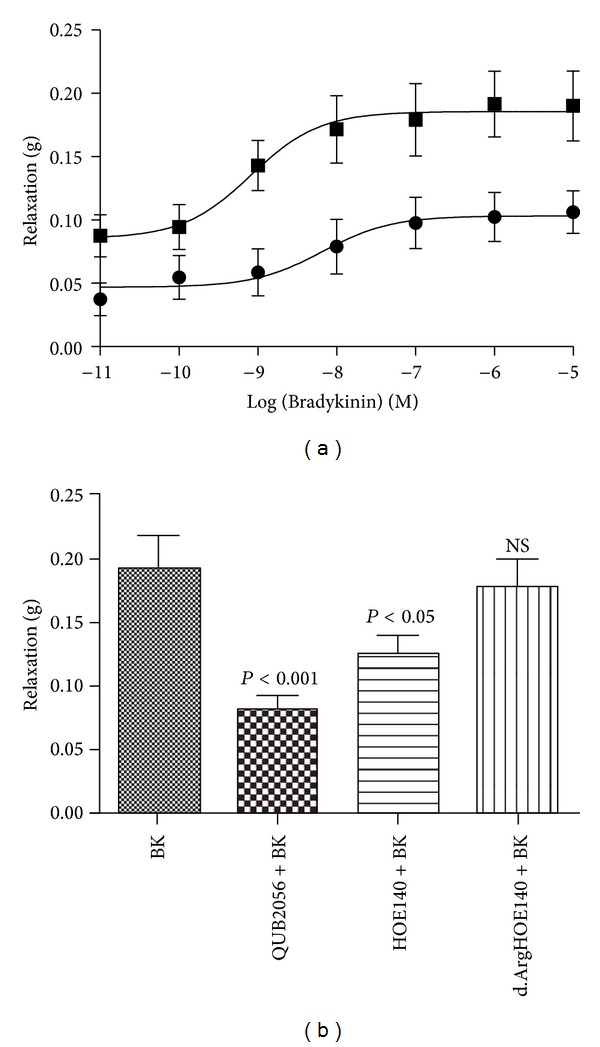
(a) Bradykinin dose-response curves using rat arterial smooth muscle in the absence (■) and presence (●) of ranakinestatin-PPF (generic name QUB2056) at a single dose of 10^−6^ M. (b) Relaxation effect of bradykinin on rat arterial smooth muscle at a single dose of 10^−6^ M and the effect of pretreatment with ranakinestatin-PPF (QUB 2056) at 10^−6^ M (*P* < 0.001), the bradykinin B_2_-receptor antagonist, HOE140 (3 × 10^−7^ M) (*P* < 0.05), and the bradykinin-B_1_-antagonist, desArg-HOE-140 (3 × 10^−7^ M) (NS—not significant). All data points represent the mean ± SEM of seven applications.

The chemical synthesis of ranakinestatin-PPF was both successful and straightforward resulting in a product of >95% purity (data not shown). The effect on the dose-dependency of bradykinin-induced relaxation of phenylephrine preconstricted rat tail artery smooth muscle was assessed following the pretreatment of preparations with a single dose (10^−6^ M) of synthetic ranakinestatin-PPF. There was a significant reduction in bradykinin-induced vasorelaxation at all concentrations between 10^−11^ M and 10^−5^ M (Figure 5(a)) (*P* < 0.05 at BK concentrations of 10^−11^ and 10^−10^ M; *P* < 0.001 at BK concentrations between 10^−9^ M and 10^−5^ M). The effects of ranakinestatin-PPF at a single dose of 10^−6^ M and the specific bradykinin B_2_- and B_1_-receptor antagonists, HOE 140, and desArg-HOE 140, at single doses of 3 × 10^−7^ M, respectively, on the vasorelaxation induced by a single dose of 10^−6^ M bradykinin blockade on rat tail artery preparations were assessed. The results (Figure 5(b)) demonstrated that the target BK receptor for the inhibitory actions of ranakinestatin-PPF was likely to be of the B_2_-subtype. The B_2_-receptor antagonist, HOE 140, caused a significant reduction (*P* < 0.05) in the vasorelaxant effects of bradykinin in this preparation with the B_1_-receptor antagonist, desArg-HOE 140, producing no significant effect. Ranakinestatin-PPF caused the most significant observed reduction in bradykinin-induced vasorelaxation (*P* < 0.001).

## 4. Discussion

Bradykinin-related peptides (BRPs) are significant components of the defensive skin secretions of many amphibians, most notably those of ranid and phyllomedusine frogs and bombinid toads [9–15, 17, 18, 20]. One of the reasons for their widespread occurrence and relative abundance is most likely due to their possession of a plethora of biological effects including those on the cardiovascular, gastrointestinal, and innate immune systems and in nociception [10, 11].

As vertebrate bradykinins are one of the few families of endogenous regulatory peptides that are taxon specific with respect to structural/functional attributes, it is not surprising that the spectrum of BRPs present in amphibian skin defensive secretions should reflect the spectrum of endogenous homologs present in their vertebrate predators [10, 11]. However, while most research has been focused on the identification of amphibian skin-derived bradykinin receptor agonists that could cause pharmacological overloads of respective predator kinin-regulated systems, there is no particular reason why natural selection should necessarily favour the evolution of agonists over antagonists. Indeed, the peptide, kinestatin, from the skin secretion of the giant fire-bellied toad,* Bombina maxima*, was found to be an antagonist of high potency on mammalian bradykinin B_2_-receptors [15].

Here, we report the prototype of another structural class of bradykinin B_2_-receptor antagonist from the skin secretion of a Chinese ranid frog,* Pelophylax plancyi fukienensis*, which was named in accordance as ranakinestatin-PPF. This peptide, unlike kinestatin, does not display any primary structural similarity to canonical bradykinin. Peptides exhibiting high degrees of identity with ranakinestatin-PPF have been previously reported from either the skin or brain tissues of the ranid frogs,* Amolops mantzorum* and* Odorrana grahami*, respectively [20]. However, the* Amolops mantzorum* skin peptide, which was named, mantzorumin-B_1_, was believed to be an antimicrobial peptide. However, the details contained under its accession number, ADM34242, indicate that the data remains unpublished and that the peptide was predicted from translated cloned precursor-encoding cDNA rather than being isolated and biologically characterized. The peptide thus has not, according to the current literature, been evaluated for antimicrobial activity and the probability is that it was named as such due to the similarity of the signal peptide and acidic spacer peptide regions of the biosynthetic precursor, with corresponding domains in the precursors of antimicrobial peptides. This, however, is a highly unreliable characteristic through which to functionally name mature amphibian skin peptides without recourse to biological evaluation. Ranakinestatin-PPF was found to be devoid of any antimicrobial activity at concentrations upto and including 250 *μ*M (2.5 × 10^−4^ M) (data not shown).

The peptide from the brain of* Odorrana grahami* was curiously named, odorranaopin, due to its antinociceptive activity despite lacking any degree of structural identity with any established opiate receptor ligand [20]. As the peptide was found to inhibit bradykinin-induced contraction of ileum smooth muscle and the fact that bradykinin is a potent nociceptive agent, it could be concluded that the antinociceptive effects of odorranaopin were mediated through its action as a bradykinin receptor antagonist rather than through any interaction with opiate receptor pathways [11, 20]. Indeed, in the present study, using rat tail arterial smooth muscle, the analog of this peptide, here more appropriately named, ranakinestatin-PPF, was found to inhibit bradykinin induced relaxation known to be mediated through the B_2_-receptor subtype [15].

Odorranaopin was predicted from a cDNA that was cloned from a brain-derived library of the frog,* Odorrana grahami* [20]. Curiously in this report, the frogs were euthanized using a technique that was very similar to that used to induce and acquire defensive skin secretions in previous reports [21]. Bearing in mind that both skin secretion peptides and their precursor-encoding cDNAs are present in such skin secretions [20, 21], it would be most difficult to acquire brain tissues from cadavers without contaminating such from this source. Thus, the possibility of a skin origin of this material was tested using skin secretion from a closely related species of frog,* Odorrana schmackeri*. Figures 4(a)–4(c) show that the cDNA encoding the peptide precursor was successfully cloned from this source indicating that in the* Odorrana* genus this is a defensive skin secretion peptide.

In view of the inconsistencies of nomenclature, we propose the name for this family of ranid frog skin bradykinin B_2_-receptor antagonists as ranakinestatins, with a suffix appended to indicate the species of origin (see Figure 4(c)). Interrogation of online protein databases with the primary structure of ranakinestatin-PPF, in addition to revealing the two previously mentioned ranid frog homologs, also located a nine-amino acid residue sequence corresponding to the N-terminal fully conserved nonapeptide of ranakinestatins, within an N-terminally located domain of the skin kininogen-2 precursor from the bombinid toad,* Bombina maxima* [15] (Figure 4(d)). Of special note is that this skin kininogen also encodes single copies of the bradykinin B_2_-receptor agonist, maximakinin (syn. bombinakinin M) [14] and the bradykinin B_2_-receptor antagonist, kinestatin [15]. The complete conservation of the N-terminal nonapeptide sequence of the ranakinestatins and its occurrence within skin kininogen-2 of a bombinid toad would suggest that this represents the active core of the peptide and structure/activity studies to address this question are underway at present.

## Data Deposition

The nucleotide sequences of cloned cDNAs encoding the biosynthetic precursors of ranakinestatin-PPF and ranakinestatin-OS, from the skin secretions of* Pelophylax plancyi fukienensis* and* Odorrana schmackeri*, respectively, have been deposited in the EMBL Nucleotide Sequence Database under the accession codes HG518554 and HG518555.
